# Management of patients with ventriculoperitoneal shunts in breast surgeries

**DOI:** 10.1016/j.jpra.2025.01.022

**Published:** 2025-02-01

**Authors:** Osama Darras, Sara Yacoub, Diwakar Phuyal, Steven Bernard, Raffi Gurunian, Sarah N. Bishop

**Affiliations:** a Department of Plastic Surgery, Cleveland Clinic, OH, USA; b Department of Plastic Surgery, Cleveland Clinic Abu Dhabi, Abu Dhabi, UAE

**Keywords:** Ventriculoperitoneal shunt, Breast, Breast reconstruction, Breast reduction

## Abstract

Ventriculoperitoneal (VP) shunts often pass through the anterior chest involving the breast parenchyma. Operating on patients with VP shunts necessitates extra caution during breast surgeries. We report two cases of female patients with VP shunts who underwent breast surgeries. We narrate the systematic approach to preserve the shunt as well as the role of interprofessional collaboration and imaging in treating these patients.

## Introduction

Ventriculoperitoneal (VP) shunts are cerebral shunts that drain excess cerebrospinal fluid, commonly indicated for patients who develop intracranial diseases including hydrocephalus and intracranial hypertension.^1^ The shunt is composed of a one-way valve that connects the ventricular catheter to the peritoneal catheter. The peritoneal catheter's path goes through the anterior chest, potentially in the breast parenchyma.^2^ The anatomical course of the catheter necessitates heightened caution when conducting breast surgeries.

In this paper, we present two cases of patients with pre-existing VP shunts who underwent breast surgeries. We discuss the surgical approach to ensure patient safety and maximize surgical outcomes.

## Case presentations

### Patient #1

A 24-year-old female presented for breast reduction surgery due to symptomatic gigantomastia. Her surgical history included VP shunt insertion for elevated intracranial pressure and scoliosis corrective surgery. Physical examination revealed significant breast hypertrophy with associated back pain and intertrigo. Preoperative neurosurgery consultation confirmed shunt functionality and planned for potential intraoperative neurosurgical intervention.

**Figure 1:**
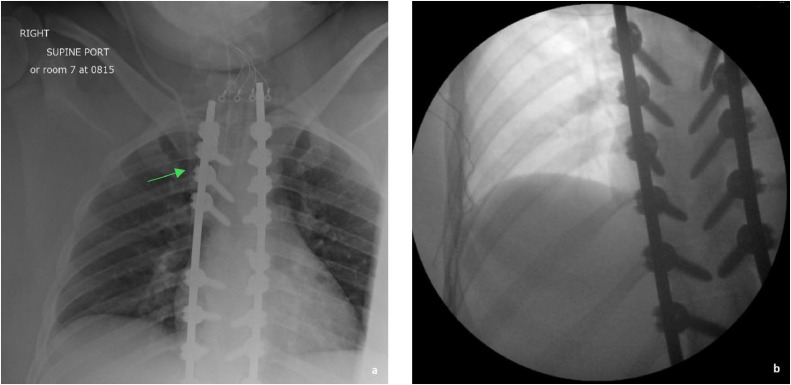
Patient #1. a- a preoperative X-ray showing VP shunt position. b- a postoperative X-ray.

On operation day, x-rays (Figure 1a) were obtained to locate the VP shunt in the chest to avoid intraoperative disturbance. A superomedial breast reduction was performed, adhering to the standard surgical technique^3^ with additional caution and time to avoid where the shunt was located. Fortunately, the shunt was not encountered during the dissection. However, if the shunt is encountered, one just needs to ensure that the shunt is not manipulated too much to avoid dislodging it. A postoperative X-ray was also obtained to confirm that the pathway of the shunt was not disturbed (Figure 1b). The patient had an uneventful postoperative course and follow-up and was content with her aesthetic and functional results, which included symptom relief The patient preserved the nipple sensation following surgery.

### Patient #2

A 20-year-old female patient, a known case of congenital Chiari malformation with hydrocephalus requiring multiple VP shunts since birth, presented with breast lumps and spontaneous nipple discharge that was later diagnosed as diffuse cystic papillomatosis, which increased her risk of developing breast malignancy.^4^ Despite offering autologous tissue reconstruction which would provide a longer-term solution, the patient opted for a bilateral mastectomy with immediate reconstruction with implants.

**Figure 2:**
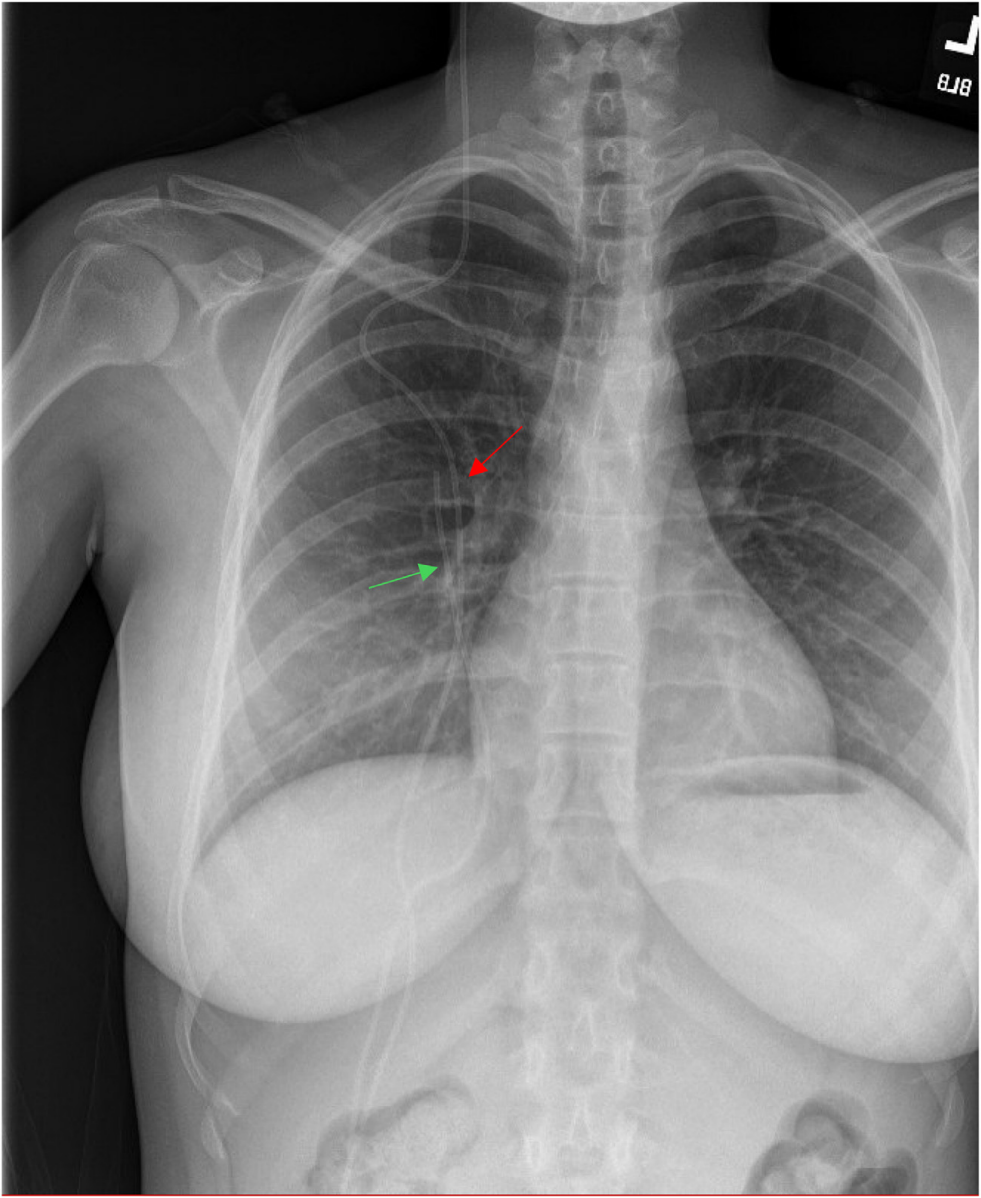
Postoperative X-ray showing the previous (green) and current (red) VP shunt positions.

Preoperative X-rays were performed to identify both previous and current VP shunts (Figure 2). During the surgery, the previous shunt fragment was removed and the current VP shunt was identified which entered the right posterior breast beneath the clavicle to the inframammary crease, and into the abdomen. A bilateral mastectomy was performed while preserving the shunt's integrity. This was followed by bilateral breast reconstruction with pre-pectoral implants and acellular dermal matrix (ADM). The patient postoperative neurosurgical outcome was unremarkable. Regarding her aesthetic outcomes, the patient requested another revision surgery for breast asymmetry.

## Discussion

Patients with VP shunts are at high risk of shunt failure, which increases with each year of shunt placement. Reported estimates include around 40 % failure after 1 year, growing to around 50 % by year 2, and 60 % by year 4.^5^ Complications related to VP shunts are common, with multiple revisions often necessary throughout a patient's lifetime. These complications can occur at any point along the tubing, including where the hardware traverses the anterior chest.

There have been reports of VP shunt complications involving pre-existing breast implants, including a case of VP shunt tubing wrapped around a breast implant, leading to a cerebrospinal fluid pseudocyst and necessitating revision of breast surgery and shunt tubing.^6^^,^^7^ Complications of breast surgery involving pre-existing VP shunts have been documented. One study implicated the dissection of a breast flap or manipulation of the medial pectoralis muscle during mastectomy as an iatrogenic cause of VP shunt puncture or damage.^8^ Another patient experienced a VP shunt fracture secondary to sub-glandular tissue expansion and implant placement, resulting in significantly increased breast swelling and symptoms of hydrocephalus. These symptoms resolved with revision surgeries of both the implant and the shunt.^9^

This paper aims to illustrate how to manage patients with VP shunts to ensure optimal outcomes for patients undergoing breast surgeries. To this end, proper pre-operative planning is critical. Planning measures include appropriate imaging to delineate the VP shunt's course. In both cases, conventional radiography was used to visualize the entire course of the shunt and assess for breaks, disconnections, or distal catheter migration.^10^ For breast reductions, one must be thoughtful as to the pedicle performed. For our patient, the pathway of the VP shunt was very medial, and we deemed that a superomedial pedicle would avoid dissecting and exposing the VP shunt. However, if the VP shunt was more lateral and centrally placed within the breast parenchyma one could potentially dislodge the VP shunt when rotating a superomedial pedicle. There is not one perfect pedicle, and the surgeon needs to take a thoughtful and tailored approach to each patient. Furthermore, an interdisciplinary approach should be employed by involving neurosurgery and ensuring their readiness to intervene as needed. In the first case, neurosurgery intervention was not needed but the team was available. However, neurosurgery intervention was required in the second case to remove the remaining fragments of the previous shunt while ensuring the patency and integrity of the shunt. Both patients were given antibiotic prophylaxis for a week following the surgery. There is no consensus over whether antibiotic prophylaxis is needed. After all, antibiotic prophylaxis should be given in case of suspected shunt contamination or proximity of the surgical site to the shunt. Regarding the second patient's choice to undergo implant-based reconstruction with an ADM, this adds a second and third foreign body. In uncomplicated cases without VP shunts, autologous tissue reconstruction decreases the probability of infection.^11^ This prevents catastrophic consequences in case an infection occurs. Implant-based reconstruction is also more prone to revision surgeries,^12^ making her a better candidate for autologous reconstruction, which would have been a better option for longevity. The patient understood these risks and opted for implant-based to avoid longer operative time and donor site morbidity. Furthermore, Imaging taken before and after the reconstruction allows the physicians to have a baseline for comparison in case a complication arises in patients with both implant-based reconstruction and VP shunts. After all, similar patients should be counseled extensively preoperatively to help them make informed decisions and understand the short-term and long-term benefits and risks of the aforementioned options in the setting of VP shunts.

## Conclusion

Breast surgeries in VP shunts are subject to unique complications that are not present in typical patients including complications involving the shunt or the breast. Intraoperative X-ray was found to be beneficial in identifying the placement of the shunt. Knowledge of the pathway of the shunt allows the surgeon to avoid damaging the shunt. We recommend neurosurgery involvement in case intervention is necessary. Collaboration is also crucial to ensure immediate corrective action in cases of VP shunt dislodgement or impending dislodgement.

## Ethical approval

Not needed.

## Declaration of competing interest

None.
